# Endoplasmic reticulum stress-induced neuronal inflammatory response and apoptosis likely plays a key role in the development of diabetic encephalopathy

**DOI:** 10.18632/oncotarget.12925

**Published:** 2016-10-26

**Authors:** Zhouguang Wang, Yan Huang, Yi Cheng, Yi Tan, Fenzan Wu, Jiamin Wu, Hongxue Shi, Hongyu Zhang, Xichong Yu, Hongchang Gao, Li Lin, Jun Cai, Jinsan Zhang, Xiaokun Li, Lu Cai, Jian Xiao

**Affiliations:** ^1^ Chinese-American Research Institute for Diabetic Complications, School of Pharmaceutical Science, Key Laboratory of Biotechnology and Pharmaceutical Engineering, Wenzhou Medical University, Wenzhou, Zhejiang, China; ^2^ Department of Pediatrics, Children's Hospital Research Institute, the University of Louisville, Louisville, Kentucky, USA

**Keywords:** ER stress, inflammation, apoptosis, p-JNK, diabetes

## Abstract

We assumed that diabetic encephalopathy (DEP) may be induced by endoplasmic reticulum (ER)-mediated inflammation and apoptosis in central nervous system. To test this notion, here we investigated the neuronal ER stress and associated inflammation and apoptosis in a type 2 diabetes model induced with high-fat diet/streptozotocin in Sprague-Dawley rats. Elevated expressions of ER stress markers, including glucose-regulated protein 78 (GRP78), activating transcription factor-6 (ATF-6), X-box binding protein-1 (XBP-1), and C/EBP homologous protein, and phosphor-Jun N-terminal kinase (p-JNK) were evident in the hippocampus CA1 of diabetic rats. These changes were also accompanied with the activation of NF-κB and the increased levels of inflammatory cytokines, tumor necrosis factor-α (TNF-α) and Interleukin-6 (IL-6). Mechanistic study with *in vitro* cultured hippocampus neurons exposed to high glucose (HG), which induced a diabetes-like effects, shown by increased ER stress, JNK and NF-κB activation, and inflammatory response. Inhibition of ER stress by 4-phenylbutyrate (4-PBA) or blockade of JNK activity by specific inhibitor or transfection of DN-JNK attenuated HG-induced inflammation and associated apoptosis. To validate the *in vitro* finding, *in vivo* application of 4-PBA resulted in a significant reduction of diabetes-induced neuronal ER stress, inflammation and cell death, leading to the prevention of DEP. These results suggest that diabetes-induced neuronal ER stress plays the critical role for diabetes-induced neuronal inflammation and cell death, leading to the development of DEP.

## INTRODUCTION

Diabetes associated cerebral atrophy and electrophysiological changes may lead to a diverse of neurological disabilities, including deficits in learning, memory, attention, as well as motor as psychomotor dysfunction. The collective term describing these disease symptoms is termed diabetic encephalopathy, DEP [1–3]. A growing body of evidence has identified that both type I and type II diabetic (T1DM and T2DM) patients exhibit a variety of neuropathological and neurobehavioral changes, including brain infarcts, cerebral white matter hyperintensities, poorer visuospatial construction, planning, visual memory, and speed [4–7]. The underlying mechanisms of DEP include, but are not limited to, elevated aldose reductase activity, advanced glycation end-product, oxidative and nitrative stress levels, inflammation, neuronal loss, and neurotropic impairment [8, 9]. Impaired insulin signaling, glucose metabolism dysregulation, and microvascular complications also contribute to the development of DEP [10, 11]. It is also appreciated that diabetes-induced changes observed in the ageing brain is associated with accelerated ageing and is the main risk factor for neurodegenerative disorders such as Alzheimer's disease [12].

Endoplasmic reticulum (ER) is an intracellular organelle known as a factory where secretory and membrane proteins are assembled. ER is also responsible for quality control of protein folding and intracellular calcium homeostasis. Errors in protein folding in the lumen of the ER can lead to accumulation of ER stress that has been traditionally viewed as an adaptive mechanism, also known as the unfolded protein response (UPR) [13]. The downstream signaling network activated by ER stress has been studied extensively. Protein misfolding is recognized by chaperones including glucose regulated protein 78 (GRP78) in the ER, where GRP78 is broken down activating pancreatic ER kinase-like ER kinase (PERK), with other transmembrane receptor proteins activating transcription factor (ATF6), and inositol-requiring enzyme 1 (IRE1) subsequently [14]. The UPR aims to remove the accumulated protein misfolding load, thus prevents any further addition of ER stress. If the protein-folding homeostasis in the ER is not restored, prolonged ER stress may activate downstream PERK-eukaryotic initiation factor 2 (eIF2)/activating transcription factor 4 (ATF4)/the CCAAT-enhancer-binding protein (C/EBP) homologous protein (CHOP)-dependent pathway, which eventually cleaves caspase 3 mediating cell death [15–16]. Previous studies have shown that cell death mediated by the CHOP-dependent ER stress is likely the mechanism underlying the pathological changes in hippocampal synapses and cognitive impairment associated with hyperglycemia [17]. One recent microarray study revealed hippocampal cells adapt to T2DM-induced prolonged ER stress with partial suppression of Xbp1 [18]; however, the detail mechanism underlying the connection between ER stress and cognitive decline remains unclear. Interestingly Lupachyk et al. found CHOP deficient mice displayed improved sciatic nerve oxidative-nitrative stress and attenuated peripheral neuropathy as compared with their wild-type (WT) littermates in the setting of diabetes, suggesting dysregulated UPR induced by prolonged ER stress is implicated in the development of diabetic peripheral neuropathy [19]. Therefore, we hypothesize that ER stress may play a key role in the pathogenesis of DEP. However, most of previous studies have mainly used T1DM animal models [20, 21]. Since two types of diabetes have significant difference for the pathogenic factors to the peripheral tissues, whether the ER stress plays the universal role in the development of DEP, and what is the mechanisms by which ER stress initiates the development of DEP all remain incompletely understood.

In this study, therefore, we aimed at examining the role of ER stress-related inflammatory response and cell death in the development of DEP in a T2DM model since this is the major type of diabetes in the adult population. To these ends, we have established a T2DM rat model by high-fat diet (HFD) feeding with a single small dose of streptozotocin (STZ). We found the induction of ER stress, inflammatory responses and apoptotic cell death in the hippocampal neurons of T2DM rats that also showed the symptom of DEP. For mechanistic studies, we have applied *in vitro* cultured hippocampal neurons exposed to high glucose (HG) with and without inhibition of either ER stress or JNK. We found that diabetes-induced neuronal ER stress plays the critical role in DEP development.

## RESULTS

### T2DM led to neurons apoptosis and cognitive deficits

In our study, during the 8-week feeding period with ND or HFD (before STZ at W0, Supplemental Figure 1B), the HFD-fed rats gained body-weight at higher rates than the ND-fed rats without a significant increase of fasting plasma glucose (Supplement Figure 1C). However, after STZ treatment, their fasting plasma glucose levels was significantly increased (from W0 to W8, Supplemental Figure 1C) and body-weight was gradually decreased even though the rats remained with HFD feeding (Supplement Figure 1B). Plasma insulin resistance levels, measured at the end of 8-week HFD before STZ injection, were significantly increased in HFD/STZ (T2D) group as compared with the control group (W0, Supplement Figure 1D). In addition, the homeostasis model assessment-insulin resistance (HOMA-IR) increased, indicating the establishment of insulin induced by 8-week HFD feeding (Supplemental Figure 1E). Sixteen HFD-fed rats with an injection of STZ at the 8^th^ week of HFD-feeding successfully developed into T2DM (Supplement Figure 1A).

**Figure 1:**
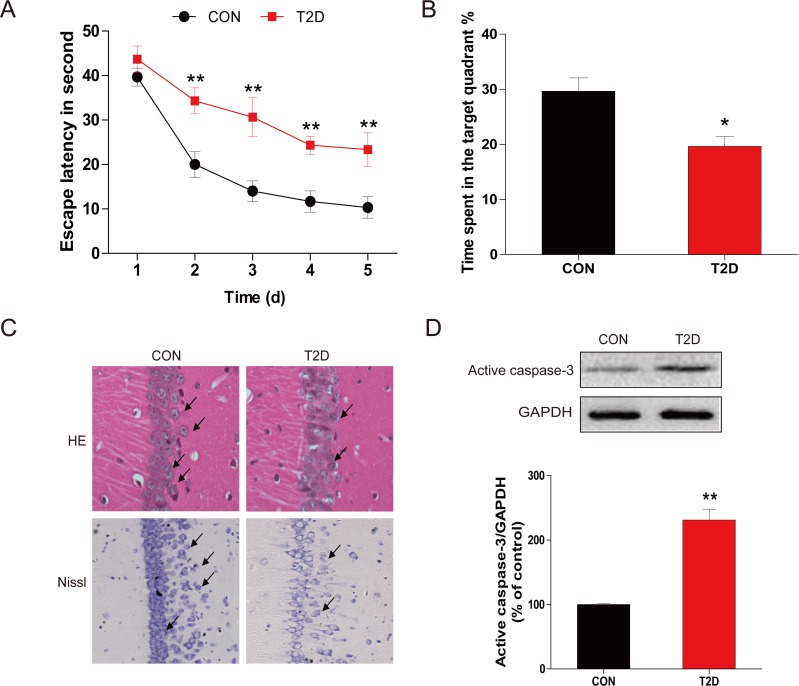
Chronic T2DM (T2D) caused cognitive deficits and neural cells apoptosis **A.** The performance of spatial memory acquisition phase 4 weeks after hyperglycemia onset in T2D and age-matched CON groups. **B.** The mean percentage of time spent in the target quadrant of CON and T2D groups, in which the platform had previously been located during acquisition. **C.** The results of H&E staining and Nissl staining in the hippocampal CA1 region for the CON and T2D groups. **D.** Western blot analysis to determine the expression of active caspase-3 in the hippocampal CA1 region of CON and T2D rats. Data were presented as mean ± SD from 8 mice in each group (*n* = 8). *P < 0.05, **P < 0.01 *versus* the CON group.

The MWM test was used to evaluate learning and memory impairment. As compared to the control group, the escape latency of the trained rats was shortened over the course of the learning trial in diabetic group (Figure 1A). When assess the learning and memory capacity in the probe trail of the MMM test, diabetic rats showed reduced percentage of time spent in the target quadrant relative to the control group (Figure 1B), suggesting the impairment of memory.

General structural examination with H&E staining (Figure 1C) revealed that healthy neurons exhibited round and pale stained nuclei that were predominantly seen in the control group. This was in contrast to dying neurons, which exhibited pyknotic nuclei were seen only in the CA1 regions of the hippocampus in diabetic rats. To evaluate diabetes-induced hippocampal neuron loss and apoptosis, histological examination of Nissl staining was performed to assess diabetes-induced neuronal loss (Figure 1C). It was found that most neurons have shrunk phenotype and were irregularly scattered in the hippocampal CA1 subfield of diabetic rats. A majority amount of neurons displayed weak Nissl staining, indicates that extensive cell death occurs. Furthermore, decreased number of surviving neuron was observed in diabetic rats as compared to their control littermates.

To further evaluate the neuronal apoptosis in hippocampus induced by T2DM, total proteins were extracted from hippocampal region. Western blot analysis for the expression of caspase-3 cleavage revealed a significant increase in diabetic group compared to controls (Figures 1D). The results of H&E staining, Nissl staining, and cleaved caspase-3 protein expression confirmed the hypothesis that the cognitive deficits induced by late-stage T2DM may associate with the apoptotic loss of hippocampal neurons.

### Hippocampal neuron apoptosis may relate ER stress and inflammatory response in HFD/STZ-induced T2DM

**Figure 2:**
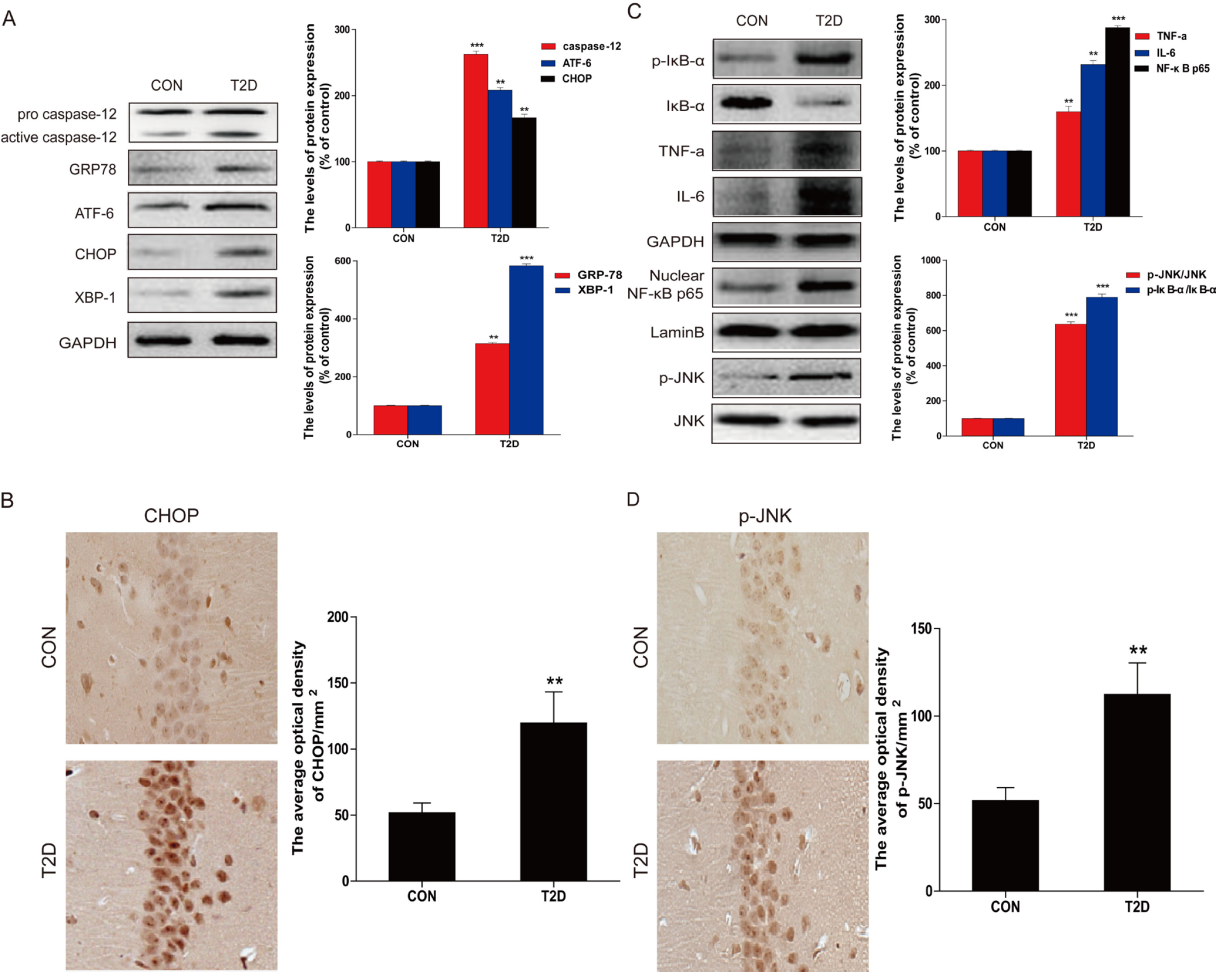
Hippocampal ER stress and inflammatory response were involved in T2D **A.** Protein extracts prepared from hippocampal region cells were subjected to immunoblotting to determine the expression of caspase-12, ATF-6, GRP78, CHOP, and XBP-1 in the hippocampal CA1 region of CON and T2D rats, and the optical density analysis of caspase-12, ATF-6, GRP78, CHOP, and XBP-1. **B.** Western blot analysis to determine the expression of p-IκB, IκBα, TNFα, IL-6 and NF-κB (p65) in the hippocampal region. **C.** Immunohistochemistry analysis for CHOP in hippocampal CA1 region of CON and T2D rats group. And the quantitative analysis of relative amount of CHOP in hippocampal CA1 region. **D.** Immunohistochemistry analysis for p-JNK in hippocampal CA1 region of CON and T2D rats group. Data were presented as mean ± SD (*n* = 8). *P < 0.05, **P < 0.01, ***P < 0.001 *versus* the CON group.

To determine whether the ER stress and inflammation response were involved in T2DM-induced hippocampal neuron apoptosis, we measured the expression of ER stress and inflammatory response related proteins with western blot. Protein levels of CHOP, GRP-78, ATF-6, XBP-1, and cleaved caspase-12 were significantly upregulated in the hippocampus of diabetic rats compared to the control group (Figure 2A). The expression of CHOP was also determined by immunohistochemistry analysis (Figure 2B). Consistent with the western blot results, few cells stained positive for ER stress-related protein CHOP in the neurons in the hippocampus of the control group, but in the diabetic group, increased amount of CHOP-expressing neurons was observed in the same region.

The expressions of inflammatory response related proteins p-IκB, IκB-α, TNF-α, IL-6, and NF-κB p65 were also measured. The inflammatory response proteins were significantly increased in the hippocampus of diabetic rats relative to the non-diabetic group (Figure 2C). These results confirmed the association of diabetes-induced hippocampal neurons apoptosis with ER stress and inflammatory response.

Considered that JNK activation plays a critical role in diabetes-induced inflammation during the development of diabetic complications [21], we have performed the immunohistochemical staining of p-JNK in the brain of control and diabetic rats, which showed a strong staining in the hippocampus neurons of diabetic mice compared to controls (Figure 2D).

### HG-induced primary hippocampus neurons apoptosis was mediated by ER stress and related inflammation

**Figure 3:**
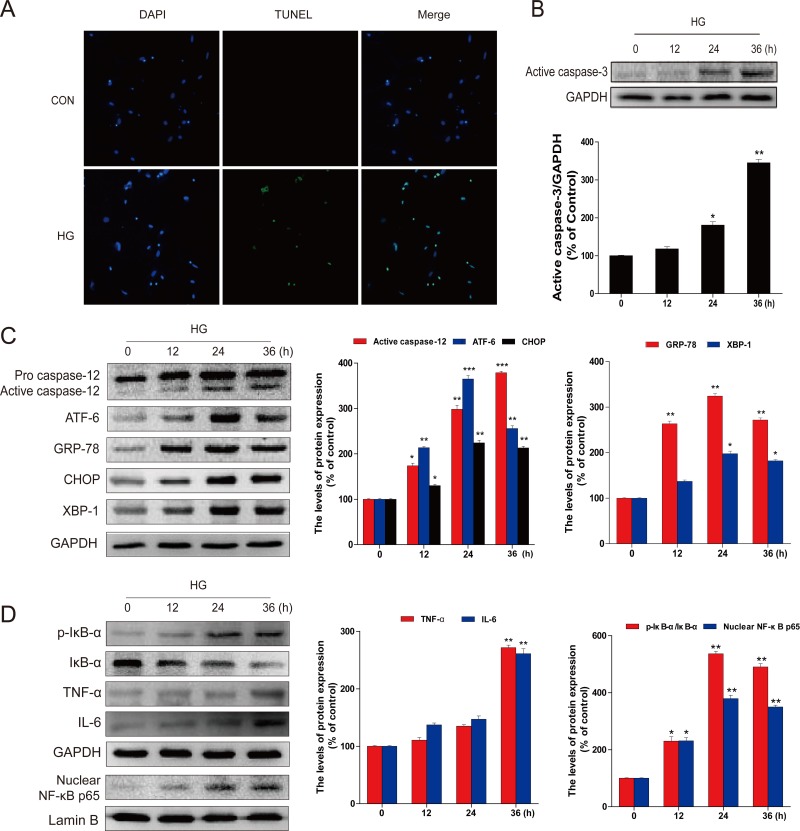
HG-induced apoptosis, ER stress, and inflammatory response in primary hippocampal neurons **A.** TUNEL assay was performed to assess apoptosis induction in the primary hippocampal neurons after high glucose (HG) treatment for 36 hours. **B.** Followed by incubation with HG (52.5 mM *vs* LG: 30.5 mM) for different time points (0, 12, 24 or 36 hours), the levels of activated caspase-3 in total lysates were determined by western blot analysis. The column figures show the normalized optical density from the data more than 3 independent experiments. **C.** Immunoblot and optical density analysis for the expression of caspase-12, ATF-6, GRP-78, CHOP, and XBP-1 in primary hippocampal neurons after HG treatment for the indicated time periods. **D.** The protein expression of TNFα, IL-6, p-IκB/IκBα, and NF-κB (p65) in primary hippocampal neurons after incubation with HG for the indicated time periods. Data were presented as mean ± SD of three independent experiments with duplicate samples at least for each condition in each experiment. *P < 0.05, **P < 0.01, ***P < 0.001 *versus* the 0 hour group.

To mechanistically investigate the roles of ER and associated inflammation in diabetes-induced hippocampus neuronal cell death, we established an *in vitro* diabetes-mimic model with primary hippocampus neurons exposed to HG (27.5 mM) in addition of medium containing 25 mM glucose (total 52.5 mM), as previously reported [22]. TUNEL assay showed a significant increase of TUNEL-positive neurons at 36 h after HG treatment (Figure 3A), along with a gradual increase of the active caspase-3 from 12 h to 36 h (Figure 3B). We also demonstrated significant increases of ER stress and inflammation responses in HG-induced neuronal cells, marked as increased expressions of cleaved caspase-12 and CHOP, as index of ER stress related pro-apoptotic mediators, and ATF-6, GRP-78 and XBP-1, as index of ER stress (Figure 3C), as well as increased expressions of p-IκB, IκB-α, TNF-α, IL-6, and p65, as index of inflammatory responses (Figure 3D). Collectively, HG-exposed primary culture of primary hippocampal neurons also recaptured what we have found in animal models (Figures 1–2), shown by increased hippocampal apoptosis, inflammation, and RE stress.

### HG-induced ER stress is the causative of the inflammatory response and cell apoptosis

**Figure 4:**
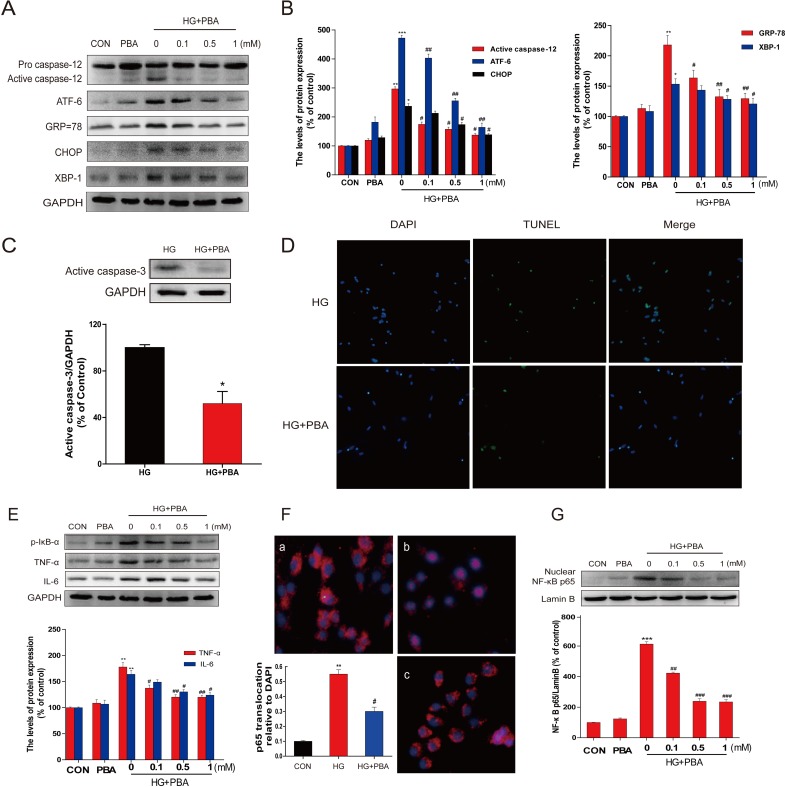
PBA administration reduced the HG-induced apoptosis, ER stress, and inflammatory response in primary hippocampal neurons **A.**-**B.** Immunoblot analysis of the caspase-12, ATF-6, GRP78, CHOP, and XBP-1 proteins expression in primary hippocampal neurons following incubation with HG and indicated doses of PBA (0, 0.1, 0.5 or 1 mM). **C.** The level of activated caspase-3 in total lysates was determined by western blot analysis. **D.** Cells were treated with HG or HG with PBA for 36 hours, TUNEL assay was performed to detect the anti-apoptosis effect of PBA in the primary hippocampal neurons. **E.** Primary hippocampal neurons were incubation with HG for 36 hours compound with different doses of PBA. Total protein was extracted and TNFα, IL-6, p-IκB/IκBα, and NF-κB (p65) proteins were subjected to western blot analysis. **F.**-**G.** An immunofluorescence-labeled staining and western blot for NF-κB p65 translocation in primary hippocampal neurons after incubated with HG and different doses of PBA for 36 hours. Data were presented as mean ± SD of three independent experiments with duplicate samples at least for each condition in each experiment. *P < 0.05, **P < 0.01, ***P < 0.001 *versus* the CON group, #*P* < 0.05, ##*P* < 0.01 *versus* the HG treatment group.

To evaluate the role of ER stress in HG-induced cell death, HG-treated primary cultures of hippocampal neurons were treated with and without 4-PBA, a specific ER stress inhibitor, for 36 h to inhibit the induction of ER stress. Western blot indicated that GRP-78, ATF-6, and XBP-1 (the UPR pathway), as well as CHOP and cleaved caspase-12 (the ER-stress associated cell death signaling molecules), were dose-dependently attenuated by 4-PBA in the group with HG/4-PBA group compared to the group with HG-exposure only (Figure 4A, 4B).

In the line of CHOP and cleaved caspase-12 changes, HG-induced cleaved-caspase-3, detected by western blot (Figure 4C), and TUNEL positive cells (Figure 4D) were also significantly suppressed by 4-PBA treatment, suggesting the prevention of cell apoptosis by the inhibition of HG-induced ER stress with 4-PBA. These results suggested that 4-PBA inhibition of ER stress is able to prevent HG-induced apoptotic loss of primary hippocampus neurons.

Next study was to define whether inhibition of HG-induced ER stress is also able to prevent HG-induced inflammation in the primary cultures of hippocampal neurons. Western blot revealed that 4-PBA alone had no effect on the expression of the inflammatory proteins p-IκB, TNF-α, and IL-6, but could dose-dependently attenuate HG-increased expression of these proteins (Figure 4E). Further analysis of NF-κB p65 by immunofluorescence staining and Western blot for nuclear protein showed that 4-PBA treatment markedly reduced the amount of HG-accelerated p65 nuclear translocation (Figure 4F, 4G). In sum, these results demonstrate that inhibition of HG-induced ER stress by 4-PBA could result in the suppression of HG-induced both apoptotic effect and inflammatory response in primary hippocampus neurons.

### HG-induced ER stress activates JNK-mediated NF-κB inflammatory signaling

**Figure 5:**
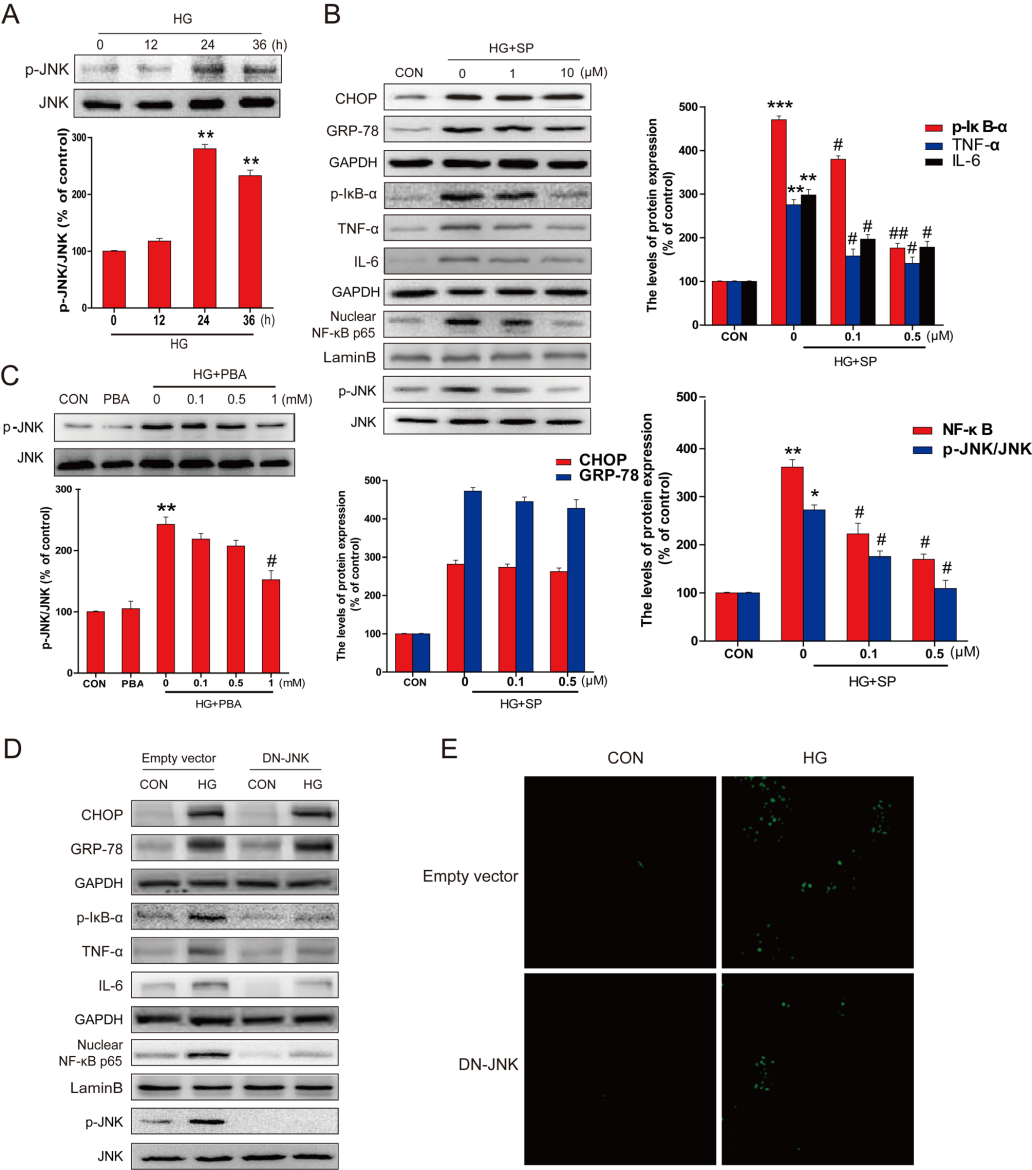
PBA administration inhibited the HG-induced p-JNK activation which was required for HG-induced inflammatory protein expression **A.** Western blot analysis for the expression and optical density analysis of p-JNK in primary hippocampal neurons after incubated with HG for the indicated period of time (0, 12, 24 or 36 hours). **B.** The expression of GRP78, CHOP, TNF-α, IL-6, NF-κB (p65), p-IκB, p-JNK and JNK in primary hippocampal neurons after incubated with HG and different doses of SP600125 (0, 1, or 10 μM). **C.** The expression and optical density analysis of p-JNK in primary hippocampal neurons after incubated with HG lone or in combination with indicated doses of PBA (0, 0.1, 0.5 or 1 mM). **D.** The expression of GRP78, CHOP, TNF-a, IL-6, NF-κB (p65), p-IκB, p-JNK and JNK in primary hippocampal neurons transferred with DN-JNK or vector after incubated with HG. **E.** The DN-JNK reduced the HG-induced primary hippocampal neurons apoptosis, when neurons were transferred with DN-JNK plasmid or vector for 24 hours before the incubation with HG for 36 hours. Data were presented as mean ± SD of three independent experiments with duplicate samples at least for each condition in each experiment. *P < 0.05, **P < 0.01, ***P < 0.001 *versus* the CON group, #*P* < 0.05, ##*P* < 0.01 *versus* the HG treatment group.

We have indicated the critical role of activated JNK in HG-induced inflammatory pathways [22]. Here the levels of p-JNK and JNK were measured by western blot, showing that the expression of p-JNK was significantly increased from 24 hours to 36 hours following HG treatment (Figure 5A). To see whether JNK phosphorylation plays a critical role in HG-induced inflammation, the primary cultures of hippocampal neurons with HG were treated with and without JNK inhibitor SP600125 for 36 hours. SP600125 dose-dependently inhibited the phosphorylation level of JNK in response to HG (bottom of the gel, Figure 5B), but had no effect on the expression of the ER stress related proteins GRP-78 and CHOP (top part of the gel, Figure 5B). However, SP600125 significantly inhibited HG-induced inflammatory response in the primary cultures of hippocampal neurons, reflected by the increased expression of p-IκB, TNF-α, IL-6, and NF-κB p65 (Figure 5B). This suggests that JNK activation plays a critical role in HG-induced inflammatory response in hippocampus cells, but did not affect its up-stream ER stress. This notion was confirmed by the fact that 4-PBA could dose-dependently suppress HG-induced JNK phosphorylation (Figure 5C).

The specificity of the pharmacological inhibitor was further assessed by *in vitro* studies. Primary neurons were transfected with a DN-JNK plasmid and then exposed to HG. Our findings demonstrated that DN-JNK could significantly attenuated HG-induced phosphorylation of JNK (Figure 5D) and the phosphorylation of IκB, TNF-α, IL-6 (Figure 5D), further confirming the function of JNK in regulating the activation of NF-κB. However, DN-JNK could not change the expression of ER stress associate protein (Figure 5D). More importantly, down-regulating JNK-phosphorylation mediated NF-κB inflammation by DN-JNK also significantly prevented HG-induced neuron apoptosis, as confirmed by TUNEL assay (Figure 5E).

**Figure 6:**
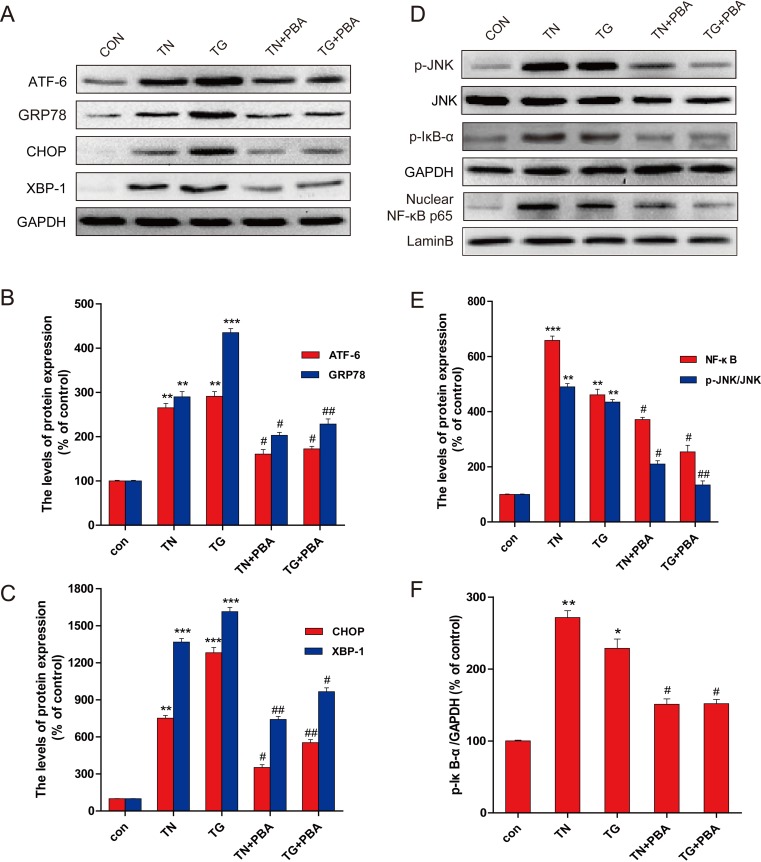
PBA administration inhibited the expression of ER-stress and inflammatory protein in the primary hippocampal neurons which induced by thapsigargin (TG) and tumicamycin (TN) **A.** Western blot expression of ATF-6, GRP78, CHOP, XBP-1 in primary hippocampal neurons after treated with TG, TN alone or with PBA. **B.** The optical density analysis of ATF-6, GRP78. **C.** The optical density analysis of CHOP, XBP-1. **D.** The expression of p-JNK, p-IκB and NF-κB (p65) in primary hippocampal neurons after treated with TG, TN alone or with PBA. (E-F). The optical density of p-JNK, NF-κB and p-IκB. Data were presented as mean ± SD (*n* = 8). *P < 0.05, **P < 0.01, ***P < 0.001 *versus* the sham group, #*P* < 0.05, ##*P* < 0.01 *versus* the TG, TN treatment group.

The above studies defined that in the primary neurons exposed to HG, JNK activation played a critical role in stimulating the NF-κB-mediated inflammatory responses and the apoptotic cell death, but JNK activation did not affect HG-induced ER stress. Therefore, next study was to define the critical role of ER stress in HG-induced JNK-mediated inflammatory responses, thapsigargin (TG) [23] and tunicamycin (TN), two well-established ER stress activators, were used to treat primary hippocampus neurons. As shown in Figure 6, TG and TN activated the ER stress related proteins (GRP-78, XBP-1, and ATF-6, and CHOP), along with a stimulation of JNK phosphorylation and inflammatory response (p-IκB, TNF-α, IL-6, and NF-κB p65). However, when these cells were treated with 4-PBA, ER stress was inhibited along with significant attenuation of JNK phosphorylation and inflammatory response. All of these data demonstrated that HG-induced ER stress play a critical role in HG-induced JNK activation-mediated inflammatory and apoptotic effect in primary hippocampus neurons.

### Down-regulation of ER stress by 4-PBA resulted in a significant prevention of hippocampal neuron apoptosis, inflammatory response and cognitive deficits in T2DM rats

To validate the importance of ER stress in HG-activated JNK-mediated inflammation and cell death, found in the *in vitro* studies, in the *in vivo* model, an experimental T2DM model was established as described in the study 1 above. These T2DM rats were treated with 4-PBA once diabetes was onset as shown in Supplemental Figure 2A. Treatment of diabetic rats with 4-PBA slightly improved the body weight loss compared to diabetic rats treated with saline (Supplement Figure 2B). Dynamic measurements of fasting blood glucose showed that 4-PBA treatment for 1 week had a significantly hypoglycemic effect on the diabetic rats, which lasted whole period of 4-week 4-PBA treatment (Supplement Figure 2C).

**Figure 7:**
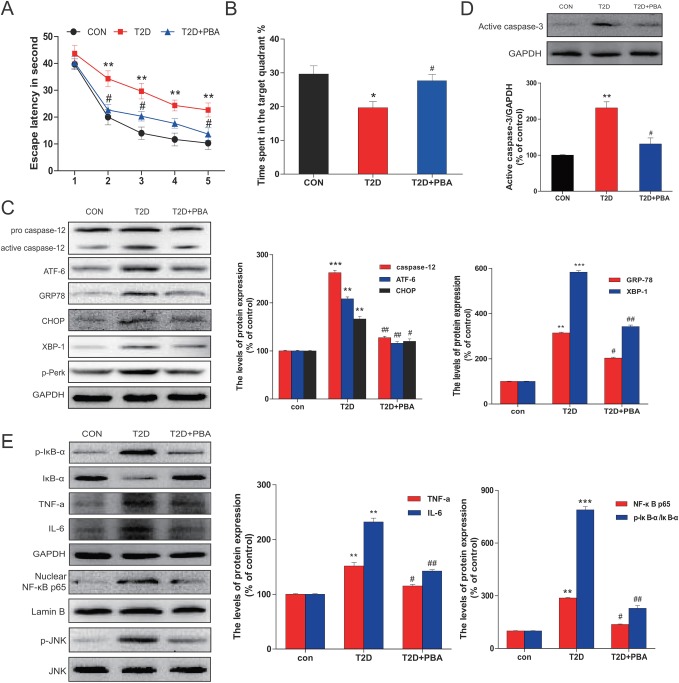
PBA administration protected chronic T2D from cognitive deficits which is associated with the neural cells apoptosis *via* inhibited the ER stress and inflammatory response **A.** The effect of PBA on the performance of spatial memory acquisition phase in T2D rats. **B.** The mean percentage of time spent in the target quadrant, in which the platform had previously been located during acquisition. **C.** Protein extracts prepared from hippocampi were subjected to immunoblotting to determine the expression of caspase-12, ATF-6, GRP78, CHOP, p-Perk and XBP-1 in the CON, T2D rats or T2D rats treated with PBA groups. **D.** Total proteins extracted from hippocampal region were subjected to western blot analysis for the determination of activated caspase-3. **E.** Western blot analysis to determine the expression of p-IκB, IκBα, TNFα, and IL-6 in the hippocampal CA1 region. The column figures show the normalized optical density from the data more than 3 independent experiments. Data were presented as mean ± SD (*n* = 16). *P < 0.05, **P < 0.01, ***P < 0.001 *versus* the CON group, #*P* < 0.05, ##*P* < 0.01 *versus* the T2D rats group.

To evaluate whether 4-PBA improves cognitive impairments observed in diabetic rats, we examined the learning and memory by the MWM test. Compared to the control rats, the escape latency was significantly long in diabetic rats throughout the learning trials. Treatment of diabetic rats with 4-PBA dramatically reduced the transfer latency to almost control levels (Figure 7A). Diabetic rats spent significantly reduced percentage of time within the target quadrant as compared to the control group, but treatment of diabetic rats with 4-PBA completely prevented diabetic effect on the spending time in the target quadrant (Figure 7B), suggesting that 4-PBA administration prevented diabetes-impaired memory.

**Figure 8:**
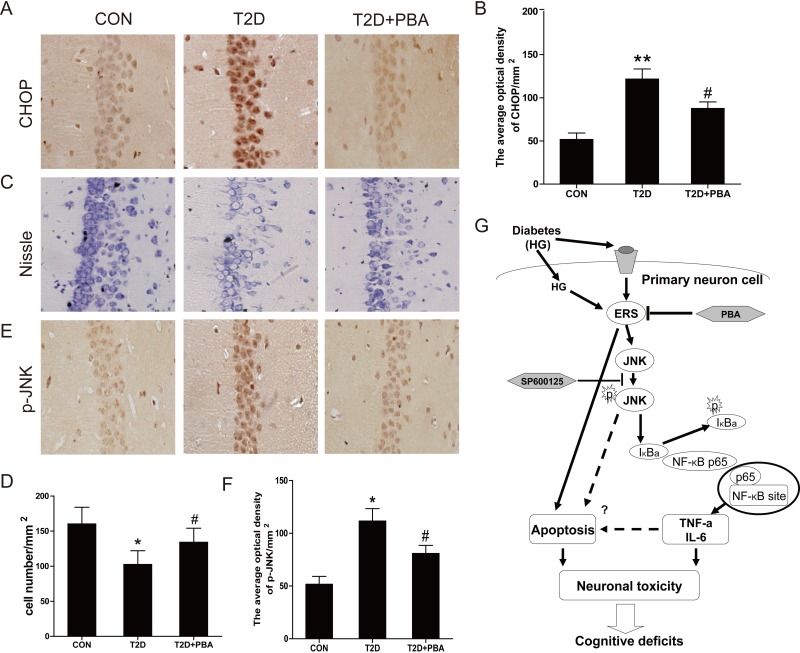
PBA administration protects the neural cells in the CA1 region of the T2D rats, and inhibits the expressions of CHOP and p-JNK protein **A.** Immunohistochemistry analysis for CHOP. **B.** Analysis of the optical density of CHOP in the hippocampal CA1 area. **C.**-**D.** The results of Nissl staining for the CON, T2D and T2D treated with PBA groups. **E.** Immunohistochemistry analysis for p-JNK. **F.** Analysis of the optical density of p-JNK protein in the hippocampal CA1 area. **G.** A schematic diagram depicting the potential molecular mechanisms underlying HG-induced cell death and PBA protection *via* alleviating ER stress and NF-κB activation. Data were presented as mean ± SD (*n* = 8). *P < 0.05, **P < 0.01 *versus* the CON group, #*P* < 0.05 *versus* the T2D group.

To further define whether the neuronal protection by 4-PBA from diabetes, ER stress and inflammatory responses were examined. Results demonstrated that treatment of 4-PBA markedly inhibited the activation of GRP-78, ATF-6, XBP-1, CHOP and cleaved caspase-12 in the hippocampus that were obviously seen in the non-treated diabetic rats (Figure 7C). Given that cell death could be mediated by activation of CHOP and caspase-12, we further applied immunohistochemical staining to localize CHOP positive cells in the hippocampus. Few CHOP-positive cells were seen in the neurons of 4-PBA-treated diabetic rats compared to diabetic rats (Figure 8A, 8B). Histological examination with Nissl staining (Figure 8C, 8D) showed that extensive amount of weakly-stained neurons were shrunken, and were arranged in smeared clusters in the hippocampal CA1 subfield of diabetic rats. When compared with the non-treated diabetic group, treatment of diabetic rats with 4-PBA significantly increased number of surviving neurons. To support the above structural and immunohistochemical examination, western blot analysis with the total proteins of hippocampal region also showed the significant activation of caspase-3 in diabetic group, but not in 4-PBA-treated diabetic rats (Figure 7D).

Considering that JNK phosphorylation as one of ER stress-mediated effects, confirmed in the above *in vitro* study, plays a critical role in the inflammatory response, we first examined the JNK phosphorylation status, by which we found that treatment of diabetic rats with 4-PBA remarkably suppressed the phosphorylation level of JNK compared to diabetic rats without treatment (Figure 7E). Corresponding to the change of p-JNK level, treatment of diabetic rats with 4-PBA also significantly prevented the activation of several inflammatory cytokines (Figure 7E). Immunohistochemical staining for p-JNK was found predominantly in the neuronal cells of hippocampus of diabetic rats, which was significantly prevented by treatment with 4-PBA (Figure 8E, 8F; Supplement Figure 3). Furthermore, a pilot study showed that *in vivo* administration of thapsigargin a chemical ER inducer exacerbates diabetic encephalopathy (Supplement Figure 4). Taken together, these results demonstrate that the neuroprotective role of 4-PBA in T2DM-induced hippocampus neurons injury is mediated by its inhibition of ER stress-activated JNK-dependent inflammatory response and nerve cells apoptosis.

## DISCUSSION

Diabetes is a chronic metabolic disorder that has become an epidemic resulting in a substantial amount of health and economic burden worldwide. Accumulating evidence has described a diverse of neurological disabilities, including cognitive impairment and memory loss, in T1DM children and long-duration T1DM and T2DM adults. It suggests that diabetes-associated neurological disabilities are secondary pathological consequences resulted by chronic glucose toxicity [24]. Prolonged exposure to high plasma glucose leads to the neuropathological complications not only in peripheral nervous system but also in central nervous systems. In addition to chronic hyperglycemia, cognitive dysfunction is also tightly associated with disorder of lipid metabolism, which occurs in T2DM [25, 26]. Reduced learning and memory capacity are likely due to the structural and functional alterations in the hippocampus and cerebral cortex [27]. Furthermore, extensive epidemiological evidence has showed that diabetes and its associated metabolic dysfunction are linked to cognitive impairment. Higher glucose level is reported to increase risk of dementia without diabetes [28], and also impaired blood glucose leads to an onset of Alzheimer's disease progressing from mild cognitive impairment [29]. However, how diabetes impacts on those brain regions and the underlying mechanisms are not well-known.

Previous animal studies have shown that both T1DM and T2DM models exhibited impaired spatial learning and memory capacity [30]. The T2DM model in this study involved a combination of a HFD to produce hyperinsulinemia and insulin resistance, followed by STZ-induced hyperglycemia. Consistent with to previous reports [31, 32], increase of body weight and high plasma level of insulin were observed after 8-week of HFD feeding. The spatial learning/memory loss and neuronal cell deaths in hippocampi were found 4 weeks after diabetes was onset, suggesting that cognitive deficits were caused by diabetes.

T2DM has been considered as an inflammatory disease [33]. Inflammatory response is necessary and protective mechanism against toxic substances produced in the brain parenchyma. In setting of diabetes, the failure of this self-contained mechanism leads to a robust of inflammation response. As a result, impaired mitochondrial function following increased production of pro-inflammatory mediators and oxidative radicals always precedes extensive neuronal damages [34, 35]. This increased level of inflammation has been attributed to the effects of poorly controlled hyperglycemia that triggers NF-κB activation and the release of pro-inflammatory cytokines [36]. In this study, we found that neuronal apoptosis and expressions of inflammatory response related proteins p-IκB, IκB-α, TNF-α, IL-6 and NF-κB p65 were increased significantly both *in vivo* and *in vitro* in the hippocampi of T2DM rats (Figure 1C, 1D, Figure 2A, 2C) as well as high-glucose treated hippocampal neurons (Figure 3A-3D), indicating that inflammation and neuronal cell death are implicated in diabetes-associated learning and memory deficits. Previous studies have related long-term insulin deficiency to a concomitant increases in tau phosphorylation and amyloid beta protein levels [37], while HFD or leptin receptor loss-of-function mutation accelerate tau pathology and spatial memory deficits in mice [38]. However, these observations were either based on chronic insulin deficient diabetic and Alzheimer disease (AD) animal models. Thus, we excluded the possibility of AD-related toxins are a prerequisite for diabetes-induced memory impairment in our animal models.

Both insulin resistance and ER stress are implicated in behavioral alternations. On one hand, insulin resistance in brains induces memory loss *via* neuronal insulin receptor substrate inhibition, as well as through mitochondrial and dopaminergic dysfunction. On the other hand, increase phosphorylation of the eukaryotic translation initiation factor 2alpha (eLF2alpha), a downstream molecule of the UPR signaling pathway, have been shown to participate in defective cognitive impairment [39]. Recent findings showed that ER stress-relevant molecules (e.g. CHOP and Xbp1) are also involved in the pathogenesis of cognitive deficits in both T1DM and T2DM models [30]. The primary function of ER is ensuring quality control of protein folding and maturation. Previous studies have showed that several pathological mechanisms, such as loss of the ER intra-luminal oxidative environment and depletion of intracellular calcium stores can disrupt homeostasis in the ER, which referred to as ER stress. ER stress has been implicated in the pathogenesis of a broad range of diseases involving accumulation of unfolded or misfolded proteins in the ER [27, 40]. Compelling evidence suggests a role for ER stress in chronic inflammatory diseases, such as inflammatory bowel diseases, diabetes, and atherosclerosis [41–44]. Recent evidence suggests that ER stress is also a possible cause of inflammation [45]. Several studies have demonstrated that ER stress up-regulated expression of inflammatory cytokines, such as TNF-α, in various cultured cell lines [46]. Increased ER stress has also been reported in the kidney of diabetic rats and contributes to inflammation and apoptosis. Recent study suggests that inhibition of endoplasmic reticulum stress pathway involving CHOP could attenuates brain ischemic injury in diabetic stroke [47]. While another research shown that the up-regulation of CHOP in hippocampal neurons of diabetic mice may promote neuronal apoptosis and account for the damaged learning and memory ability of diabetic mice [48]. However, how ER stress promotes inflammatory response and neuronal injury in diabetic hippocampi remains poorly understood. In the present study, we demonstrated that not only CHOP but also other proteins in ER stress cascade, such as GRP-78, XBP-1, ATF-6, are elevated in the hippocampi of animal models of diabetes (Figure 2A, 2B) and primary hippocampal neurons exposed to high glucose (Figure 3C). Consistently, induction of ER stress by tunicamycin or thapsigargin is sufficient to elicit inflammatory response proteins expression *in vitro* (Figure 6). Supporting this notion, inhibition of ER stress by chemical chaperone 4-PBA significantly attenuated NF-κB-activated inflammatory response and cell death in the primary cultures of hippocampal neurons exposed to high glucose (Figure 4) or RE stress activators (Figure 6). Taken together, these results provide the direct evidence that ER stress contributes to neuronal inflammation and cell death in hippocampus of T2DM rats.

Memory loss is associated with changes in the UPR network in the brain. Several lines of studies have reported that UPR activation by abnormal protein misfolding leading to phosphorylation of elF2alpha, which in turn impairs cognitive processes and synaptic function [49]. However, the most conserved signaling pathways of the UPR network is imitated by the IRE1alpha which is governed by JNK. Thus, a major question is still open in this emerging field as whether the JNK pathway is mechanistically involved in the consolidation and formation of memory. The JNK pathway plays a major role in neuronal inflammation. Activation of JNK has been observed in many brain damage models, which may be related to its critical role in mediating neuronal inflammation and neuronal cell apoptosis [50]. Recent studies show that JNK activation impairs insulin signaling transduction, resulting in diabetes-associated cognitive decline in the hippocampus of T2DM rats [51]. Accumulating ER stress promotes ATF4 directly binding to promoters of inflammatory genes and leads activation of the JNK pathway, resulting in exaggerated and prolonged inflammatory responses [52]. However, how ER stress regulates JNK signal and further mediates the inflammatory response in neurons still poorly understood. In this study, we found that treatment of HG for 24 h activated the JNK pathway in hippocampal neurons, which was abolished by incubation with 4-PBA (Figure 5A, 5C). Interestingly, treatment of SP600125 (a specific JNK inhibitor) or blocking JNK-targeted genes by DN-JNK completely prevented HG-induced inflammatory response and cell death without affecting ER stress signal pathway (Figure 5B, 5D, and 5E). Similarly, phosphorylation of JNK signaling by ER stress activator TG or TN was reversed by 4-PBA (Figure 6D, 6E). These collective findings suggest that the ER stress induced by HG triggers JNK-dependent NF-κB-mediated inflammatory pathways, both chronic apoptotic and inflammatory effect contributes the development of diabetic encephalopathy (Figure 8G).

Many therapeutic drugs such as melatonin and vitamin E have been reported to prevent diabetes animals from learning and memory deficiency [53]. The 4-PBA is commonly used to alleviate ER stress. It can interact with hydrophobic domains of mis-folded proteins and prevent their aggregation, which enhances the chances for correct folding of proteins to get their right conformation. Most importantly, the blood-brain barrier is permeable to 4-BPA. Systematic administration of 4-PBA on HFD/STZ T2DM rats significantly attenuated RE stress, JNK-dependent NF-κB-mediated inflammation, and apoptotic cell death in hippocampi, which resulted in a partial recovery of spatial learning and memory consolidation (Figure 7 and Figure 8A-8F). However, weight gain and declined hyperglycemia after 4-PBA treatment (Supplemental Figure 2B, 2C) suggested ER stress may also be suppressed to protect β-cells from STZ-induced cell death, which eventually alleviates the cognitive deficits and hippocampal damage.

In a conclusion, ER stress-mediated brain inflammation *via* activation of JNK signaling is essential for cognitive disorder in T2DM. ER stress may directly induce cell death or indirectly by inflammation. Inhibition of ER stress significantly attenuated cognitive deficits and reduced neuronal apoptosis in T2DM rats and HG-induced hippocampal neurons. Therefore, inhibition ER stress is a potential therapy of diabetes related cognitive deficits.

## MATERIALS AND METHODS

### Reagents and antibodies

4-phenylbutyrate (4-PBA) was purchased from Sigma Co. (Sigma-Aldrich, St. Louis, MO). Dulbecco's Modified Eagle Medium (DMEM), F12 and Fetal Bovine Serum (FBS) were purchased from Invitrogen (Invitrogen, Carlasbad CA). Primary antibodies against GRP78, CHOP, XBP-1, TNF-α, IL-6, caspase-12, Lamin B, GAPDH, IκB-α, NF-κB p65, ATF-6, p-JNK, JNK and IgG-HRP were purchased from Cell Signaling Technology, Inc. (Shanghai, China). A 3.3-kb cDNA fragment (dominant-negative type) encoding HA-tagged JNKK2 (DM)-JNK1 fusion protein, in which lysine 149 in the ATP domain of the JNKK2 moiety was replaced by methionine, and vector cDNA (control) were used, as previously described. Enhanced chemiluminescence (ECL) Kit was purchased from Bio-Rad (Hercules, CA). STZ, thapsigargin, tunicamycin, JNK inhibitor SP600125, and all of the other reagents were purchased from Sigma Co. and otherwise specified.

### Diabetic induction and sample collection

Adult male Sprague-Dawley rats were purchased from the Animal Center of the Chinese Academy of Sciences. Rats were maintained at room temperatures (25 ± 2°C) under a standard 12-hour/12-hour light-dark cycle with free access to water and food. All animal work was performed under protocols approved by the Institutional Animal Care and Use Committee at the Wenzhou medical University.

A total of 24 rats were included in study 1. 16 rats were subjected to HFD and 8 rats were fed with normal chow. As shown in Supplemental Figure 1, A T2DM model was established by feeding HFD (67% normal diet plus 10% lard, 20% sucrose, 2% cholesterol, and 1% sodium cholate) for 12 weeks, starting at 10-12 weeks of age. In parallel, aged-matched control group under normal chow conditions were examined [7]. Metabolic studies were performed after 8 weeks of HFD feeding. Homeostasis model assessment-insulin resistance (HOMA-IR) was calculated with the formula of HOMA-IR = FINS (mIU/L)*FPG (mM)/22.5, where FINS (fasting insulin) and FPG (fasting blood glucose) were determined by ELISA. Once insulin resistance was developed in the HFD group, an intraperitoneal injection of STZ (dissolved in 50 mM citrate buffer (pH 4.5) for three days; at a dose of 35 mg/kg body weight was performed to induce hyperglycemia in these HFD-fed rats. Three days after STZ treatment, rats with a blood glucose level higher than 16 mM/L were considered to be diabetic and were included in further experiments.

A total of 40 rats were used in Study 2. Rats were randomly divided to be fed with HFD (DM, *n* = 32) or with normal chow (ND, *n* = 8). Rats-fed with HFD were treated with STZ, as previously described in Study 1. These diabetic animals were further divided into two experimental groups: Control, treatment. The control group received an oral gavage of saline, while the treatment group received an oral gavage of 4-PBA (dissolved in saline, 1 g·kg^−1^·d^−1^) daily for 4 weeks. The 4-PBA was prepared by titrating equimolar amounts of 4-phenylbutyric acid (Sigma, USA) and sodium hydroxide to pH 7.4. Sixteen rats were used per experimental group (*n* = 16).

Body weights and blood glucose were monitored weekly. After 4-PBA treatment, animals were trained to perform learning and memory task in Morris water maze for five consecutive days. At the end of behavior studies, hippocampus was harvested for subsequent morphological and biochemical studies. Protein and RNA samples were kept at −80°C until the measurements were performed.

### Morris water maze (MWM) test

Animals were tested in a spatial version of Morris water maze test as described previously [54]. The MWM consisted of a circular water tank (120 cm diameter, 50 cm height) filled with opaque water (mixed with milk power, 25°C). Animals were allowed to acclimate to the task environment with swimming in the pool without a platform for two days. Each training session lasted for 2 minutes. Subsequently, the pool was divided, virtually, into four equal quadrants, labeled as N-S-E-W. A platform (10 cm diameter) was placed in one of the four maze quadrants (the target quadrant) and was submerged 1.5 cm beneath the surface of water. The platform was kept in the same quadrant during the entire experiment. Animals were required to locate the platform only relying on distal spatial cues available in the testing room. The cues were maintained constantly throughout the experiment. These rats received four consecutive training trials daily for five days, with each trial lasted for 60 seconds and 30 seconds in between. The rat had to swim until it was able to locate the platform that is submerged underneath the water. The animal was allowed to be remained on the platform for 30 seconds before the commencement of the next trial. The escape platform was kept in the same position relative to the distal cues. If the rat failed to locate escape platform within 60 seconds, the rat was instructed to locate the platform and was allowed to be remained there for the same amount of time. The time to locate the platform was measured as latency in second. Automated behavioral analysis was conducted using ANY-maze, which is a flexible video tracking system designed to automate testing in behavioral experiments (ANY-maze©, Stoelting Co., USA).

### Probe trial

A probe trial was performed, wherein the extent of memory consolidation of the animal was assessed. The time spent in the target quadrant indicates the degree of memory consolidation that has taken place after learning. In the probe trial, the hidden platform was removed from the pool while the distal cues were kept. The rat was allowed to swim in the pool, as previously described in the training trials. The percentage of time spent in the target platform quadrant and the number of platform crossings were recorded to measure the spatial memory retention.

### Histology and immunohistochemistry

Rats were anesthetized with 4% choral hydrate (10 ml/kg) and then perfused with 4% paraformaldehyde in 0.1 M phosphate buffer (pH 7.4). Brains were removed and post-fixed for 24 hours in the same fixative. Post-fixed brains were embedded in paraffin and sliced on a microtome at a 5 μm thickness. The sections between 2 mm and 3 mm posterior of the bregma were used for this study. For histological assessment of damage to the hippocampus, the paraffin-embedded brain sections were mounted in Poly-L-Lysine-coated slides for histopathological examination by H&E staining. The sections were also incubated in 1% cresyl violet for Nissle staining. To determine CHOP and p-JNK activities, sections were incubated with 0.3% H_2_O_2_ in methanol for 30 minutes, and then submerged into blocking solution (1% albumin bovine in PBS) for 1 hour at room temperature prior staining. Primary antibodies, such as CHOP (1:200) and p-JNK (1:500) were applied on sections with overnight incubation at 4°C. After 3 times of PBS wash, they were incubated with horseradish peroxidase-conjugated secondary antibodies for 2 hours at 37°C. The reaction was terminated with 3, 3′-diaminobenzidine, and images were captured at 400x magnification using a Nikon ECLPSE 80i. The optical density of CHOP and p-JNK within the injury site in hippocampus were counted at five randomly selected fields per sample [55].

### Primary hippocampal neurons culture and treatment

Neonatal Sprague-Dawley rats were used for primary hippocampal neuron culture studies. Hippocampi were dissected and rinsed in ice cold dissection buffer. Blood vessels and white matter were removed and hippocampi were incubated with 0.125% trypsin in Hanks' balanced salt solution at 37°C for 20 minutes. The whole solution was filtered through stainless steel (200 mesh, hole-width 95 μm). Cell suspension was centrifuged at 1000 rpm for 10 minutes twice and the remaining cell pellet was resuspended in DMEM/F-12 with 20% FBS, 100 U/l penicillin, 100 mg/l streptomycin and 0.5 mM glutamine. Cells were seeded at a density of 1 - 5 × 10^5^/ml in 6 well plates with coverslips precoated with poly-L-lysine in a 5% CO_2_ incubator at 37°C. After 24 hours, the culture medium was replaced with fresh DEME/F-12 medium every two or three days. Arabinosylcytosin (10 mg/l) was added at 72 hours to prevent the growth of non-neuronal cells. The purity of neurons was measured by staining with anti-microtubule associated protein 2 (MAP-2) antibody (1:50) using immunofluorescence staining. Cells pre-treated with different doses of 4-PBA, were incubated with HG (DMEM containing 25 mM D-glucose, HG: 25 + 27.5 = 52.5 mM glucose) or low glucose (LG: 25 + 5.5 = 30.5 mM glucose) for 12, 24 or 36 hours.

### Transient transfection

Freshly isolated hippocampal neurons were incubated in 1 mL of serum-free medium containing 10 μL of Lipofectamine 2000 reagent (Invitrogen, City, CA) and 4 μg of dominant negative JNK (DN-JNK) or vector for 6 hours with fresh medium replaced afterward. Primary neurons were treated with HG for 24 or 48 hours for further investigation.

### Western blot analysis

For protein extraction, the frozen tissue was homogenized in modified RIPA buffer (50 mM Tris-HCl, 1 % NP-40, 20 mM DTT, 150 mM NaCl, PH = 7.4) containing protease inhibitor cocktail (10 μl/ml, GE Healthcare). Samples were then centrifuged at 12,000 rpm and supernatants were extracted for protein assay [56, 57]. In contrast to frozen tissues, primary hippocampal neurons culture were lysed in RIPA buffer (25 mM Tris-HCl, pH 7.6), 150 mM NaCl, 1% Nonidet P-40, 1% sodium deoxycholate, and 0.1% SDS) with protease and phosphatase inhibitors. Protein concentrations were quantified with bicinchoninic acid reagents (Thermo, Rockford, IL). An equal amount of protein (50 μg) was fractionated by 11.5 % gel, transferred onto a PVDF membrane (Bio-Rad). Membranes were blocked with 5 % milk (Bio-Rad) in TBS with 0.05 % tween 20 for 1 hour and incubated with the primary antibodies: GRP78 (1:300), CHOP (1:300), XBP-1 (1:300), caspase-12 (1:200) TNF-α (1:200), IL-6 (1:200, p-IκB (1:1000), IκB-α (1:1000), NF-κB p65 (1:1000), ATF-6 (1:1000), p-JNK (1:1000) and JNK (1:1000), in 5 % milk in TBS with 0.05% tween 20 overnight. Membranes were washed with TBS for 3 times and treated with horseradish peroxidase- conjugated secondary antibodies for 2 hours at room temperature. Proteins were detected using (what ECL solution) and imaged by ChemiDicTM XRS+ Imaging System (BioRad). Signal intensities were densitometrically quantified by Multi Gauge Software of Science Lab 2006 (FUJIFILM Corporation, Japan).

### Terminal deoxynucleotidyl transferase-mediated dUTP-biotin nick end labeling (TUNEL) assay

DNA fragmentation in primary hippocampal neurons were detected using the one step TUNEL Apoptosis Assay KIT (Roche, Mannheim, Germany). Cultured cells were fixed with 4% paraformaldehyde at 4°C for 20 minutes. and treated with 0.3% hydrogen peroxide at room temperature for 15 minutes, and then treated with 0.2% Triton X-100 for 15 minutes. After being washed in PBS, cells were incubated with biotinylated nucleotide and the terminal deoxynucleotidyl transferase, recombinant (rTdT) enzyme at 37°C for 1 hour, then mounted with DAPI in antifade solution for 5 minutes [58]. Images were captured with a Nikon ECLIPSE Ti microscope (Nikon, Japan).

### Assay of cellular NF-κB p-65 translocation

Primary hippocampal neurons were labeled according to the manufacturer's instruction using a Cellular NF-κB p65 Translocation Kit (Beyotime Biotech, Nantong, China). The p65 protein and nuclei fluorescence are in red and blue respectively, which can be simultaneously viewed by fluorescence microscope (×200; Nikon) at an excitation wavelength of 350 nm for DAPI and 540 nm for Cy3. To create a two-color image, the red and blue images were overlaid. Experiments were performed in duplicate for at least three times.

### Statistical analysis

Data are expressed as mean ± SEM. Statistical significance was determined with Student's *t*-test if compared two experimental groups or one-way Analysis-of-variance (ANOVA) test followed by Dunnett's post hoc tests if analyzing more than two groups. Differences were considered to be statistically significant with the value *p* < 0.05 considered significant.

## SUPPLEMENTARY MATERIAL


