# Supplementary material for: Endoplasmic reticulum stress-induced neuronal inflammatory response and apoptosis likely plays a key role in the development of diabetic encephalopathy
Source: Oncotarget. 2016 Oct 26;7(48):78455–72. doi: 10.18632/oncotarget.12925 (PMC5346653; doi:10.18632/oncotarget.12925)
Supplement: Supplementary file 1 [file oncotarget-07-78455-s001.pdf]

Endoplasmic reticulum stress-induced neuronal inflammatory response and apoptosis likely plays a key role in the development of diabetic encephalopathy

Supplementary Material

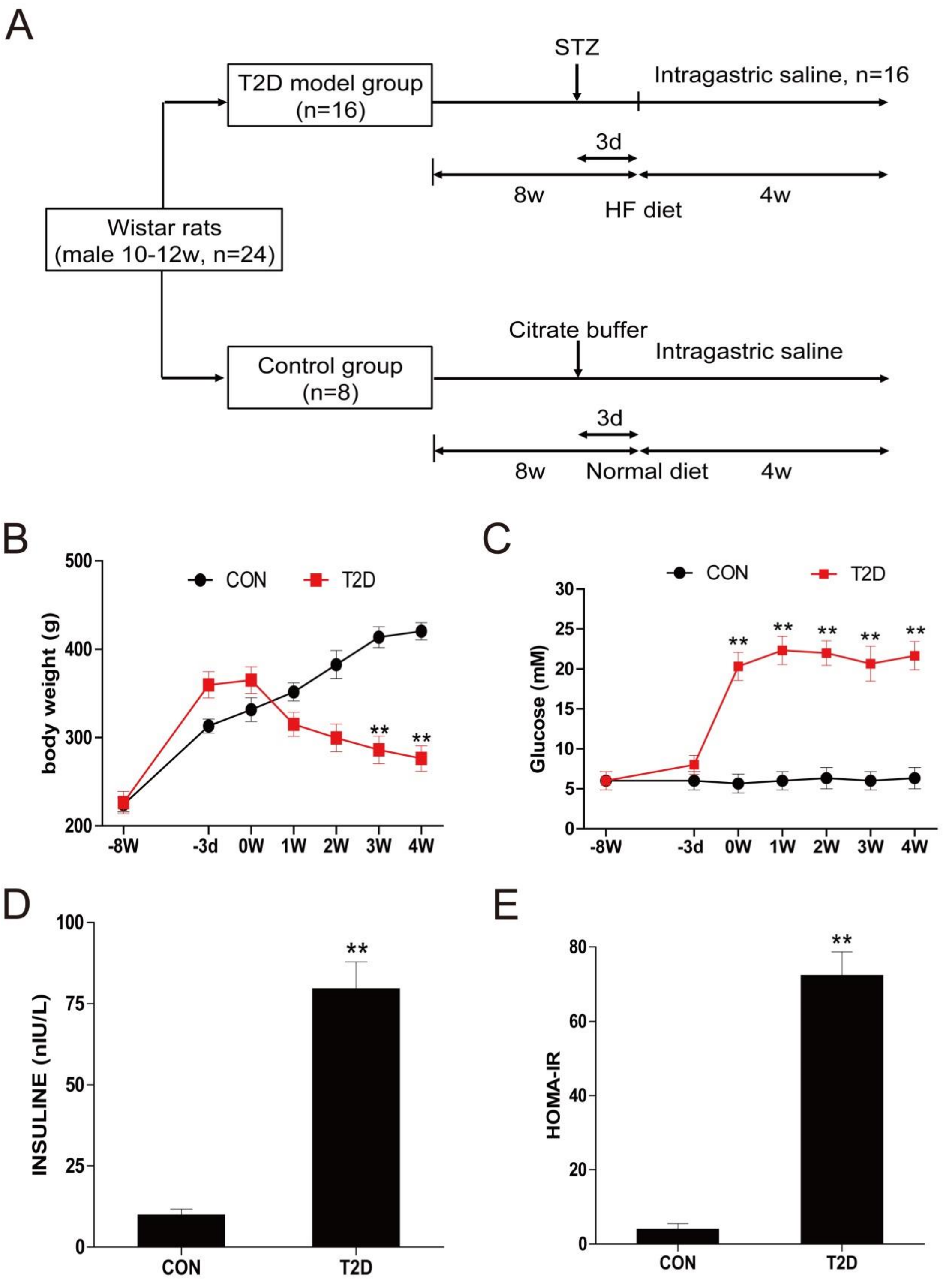

Supplement Fig. 1. Diagram of animal study design (A).The body-weight (B) and fasting plasma glucose levels (C) before and after injection of STZ. The fasting plasma insulin concentrations (D) and HOMA-IR (E) were measured just before STZ injection. **P*<0.05, ***P*<0.01 versus the CON group.

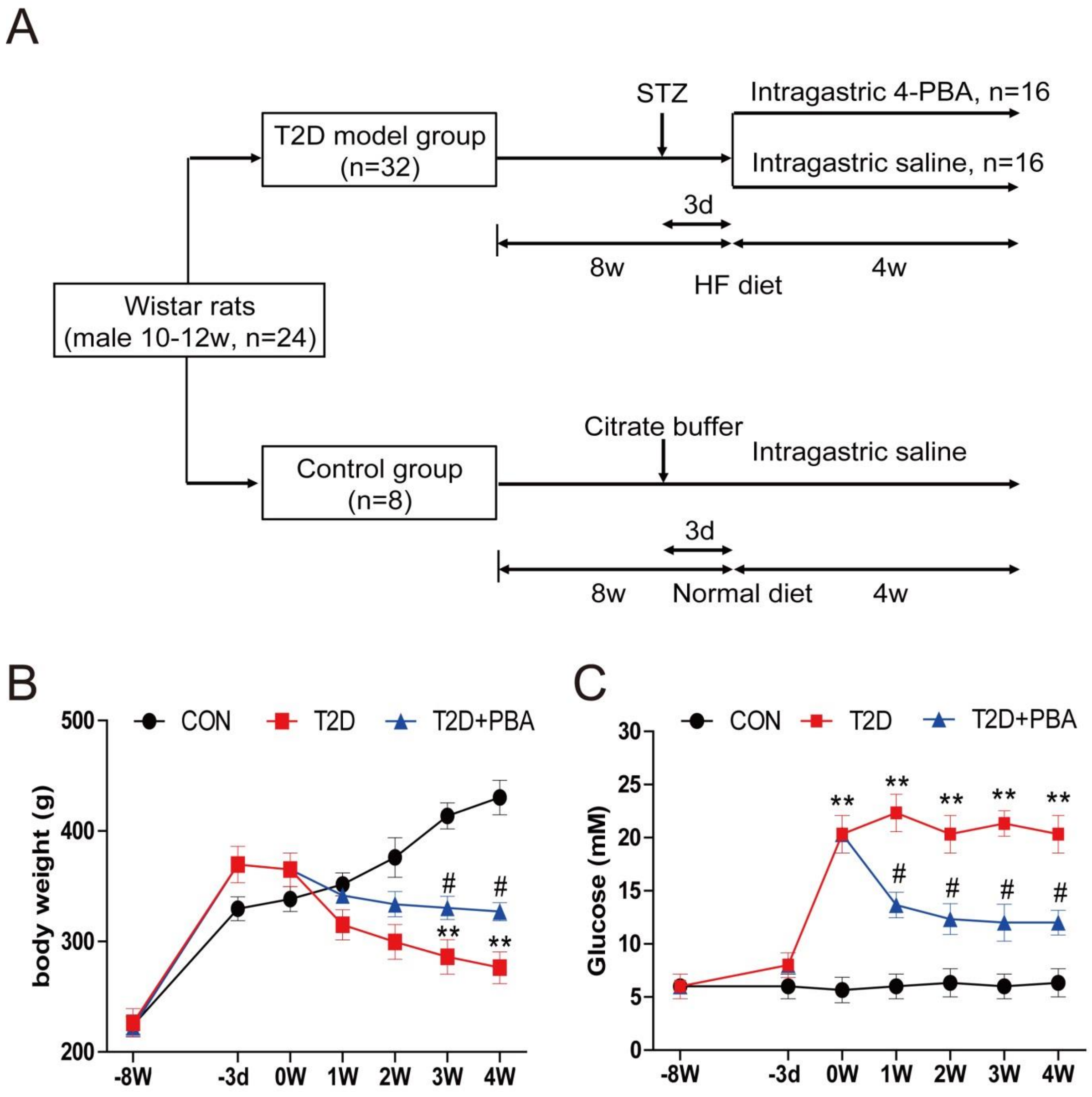

Supplement Fig. 2. Diagram of animal study design (A).The body-weight (B) and fasting plasma glucose levels (C) were dynamically measured. **P*<0.05, ***P*<0.01 versus the CON group.

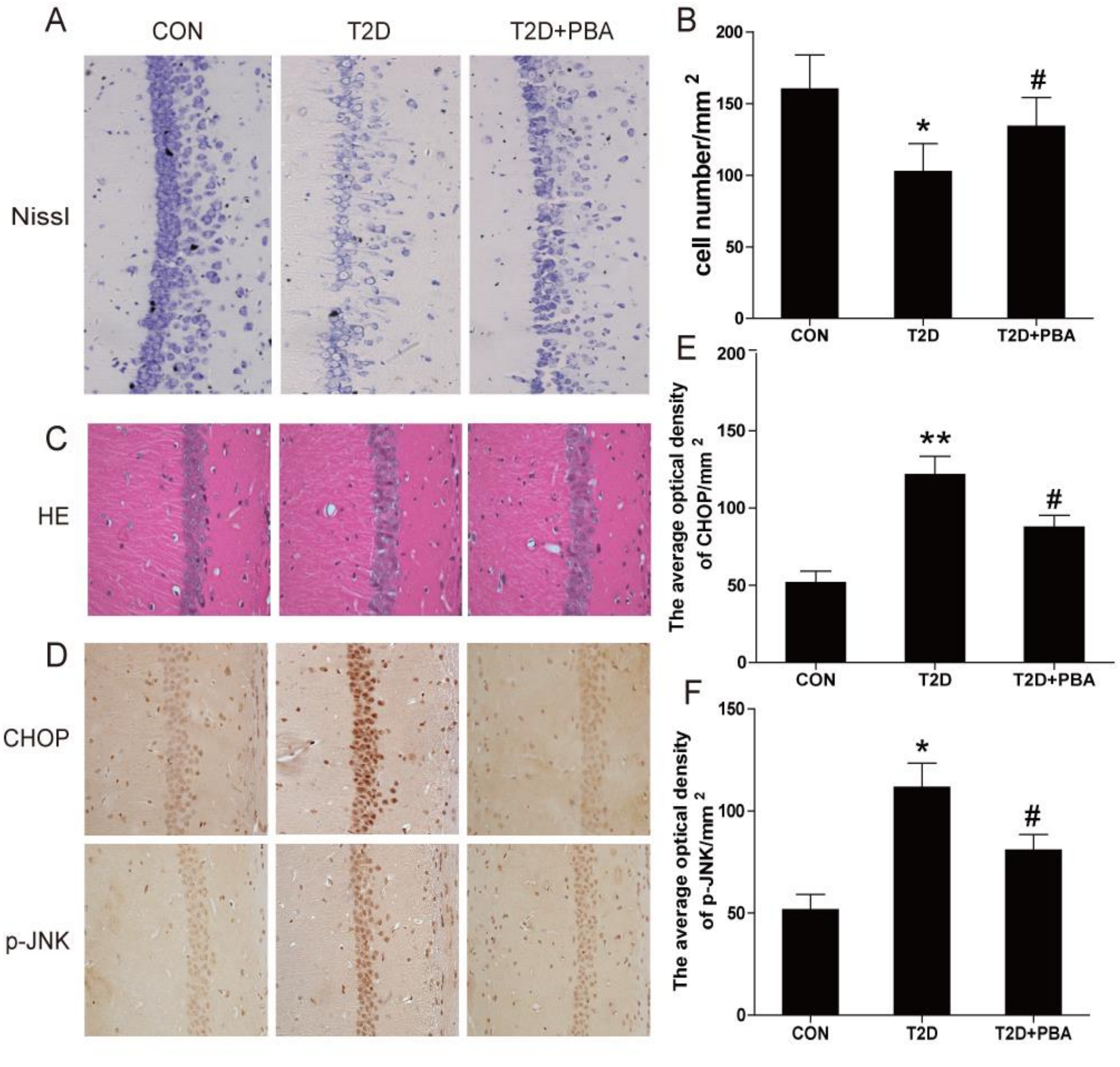

Supplement Fig 3. Low magnification of brain sections.The results of Nissl staining (A-B)and

H&E staining (C) in the hippocampal CA1 region for the CON, T2D and PBA treatment groups. (D) Immunostaining analysis to determine the expression of CHOP and p-JNK in the hippocampal CA1 region of CON, T2D and PBA treatment rats. Data were presented as mean ± SD (n=8). *P<0.05, **P<0.01 versus the CON group.

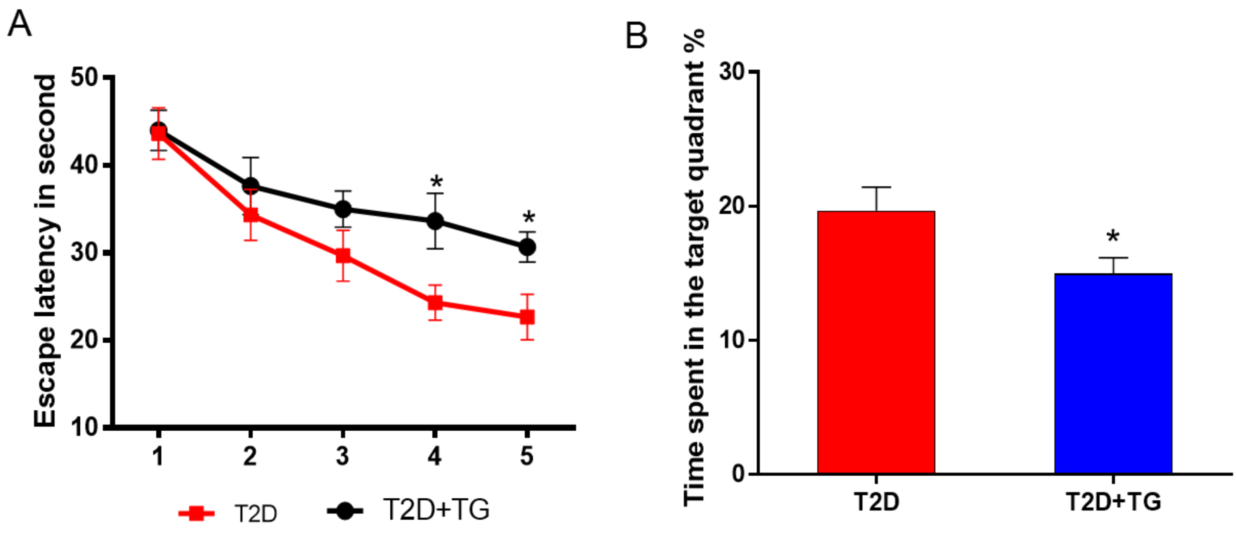

Supplement Fig. 4. TG treatment could further induce memory loss in T2D rats. (A) The performance of spatial memory acquisition phase 4 weeks after hyperglycemia onset in T2D and TG treatment groups. (B) The mean percentage of time spent in the target quadrant of T2D and TG treatment groups, in which the platform had previously been located during acquisition. *P<0.05 versus the T2D group, n=8.
